# The Unexpected Role of Carbonate Impurities in Polyphosphate Corrosion Inhibition

**DOI:** 10.1038/s41598-018-35512-2

**Published:** 2018-11-28

**Authors:** Suzanne Morsch, Seyedgholamreza Emad, Lee A. Farren, Matthew D. Goodall, Stuart B. Lyon, Simon R. Gibbon

**Affiliations:** 1 The University of Manchester, Corrosion and Protection Centre, School of Materials, Manchester, M13 9PL UK; 2 AkzoNobel, Gateshead, NE10 0JY UK

## Abstract

Polyphosphate corrosion inhibitors are increasingly marketed as chromate replacements for coil coated steel. The mechanisms underpinning corrosion prevention by these species is, however, not fully understood; corrosion inhibition is ordinarily assessed using electrochemical techniques, followed by *ex-situ* surface analysis. As a result, the formation of a clear film over cathodic sites is known to contribute to corrosion prevention, but little is known about its formation. Here, we apply advanced microscopy techniques (*in-situ* fluid cell AFM, SEM-EDX, and AFM-IR nano-chemical analysis) to examine early cathodic film formation by strontium aluminium polyphosphate (SAPP) in detail. For a model cut edge system, it is found that cathodic inhibition dominates during the first 24 hours of immersion, and surprisingly, that strontium carbonate impurities play a significant role. Rapidly precipitated zinc carbonate provides protection almost immediately after immersion, before the film structure evolves to include (poly)phosphate species. This suggests that the purposeful inclusion of carbonates may provide a new, environmentally sound approach to enhancing inhibitor efficacy.

## Introduction

For coil coated steel structures (e.g., architectural cladding), the cut edge remains a uniquely vulnerable point for the initiation of atmospheric corrosion. This susceptibility arises because at the cut edge, the steel, metallic coating and organic coating system are directly exposed to the environment. Under such conditions, the thin layers of protective zinc (galvanised steel) or zinc-aluminium alloy (e.g., galfan, galvalume) essentially behave as sacrificial anodes and can become depleted (potentially undermining the organic coating, resulting in crevice corrosion etc.)^1^. Historically however, inhibitor pigments embedded in a primer layer have provided a robust approach to mitigate corrosion at cut edges. Upon exposure to an aqueous environment, these compounds leach out and provide protection by retarding the anodic or cathodic reaction (or both). For decades, chromate pigments were incorporated into primer coatings to this end, providing unrivalled protection as a consequence of mixed anodic and cathodic inhibition^2–4^. Recently however, concerns about the toxicity of chromium (VI) compounds has culminated in increasingly strict regulation (e.g., REACH legislation) limiting their use. This has driven an intensive research effort to find effective, environmentally sound chromate replacement systems.

Polyphosphates have emerged as commercially viable chromate replacements for some applications (e.g., strontium aluminium polyphosphate, SAPP, and zinc aluminium polyphosphate, ZAPP, pigments), but their performance is not well-understood^5,6^. Simple phosphate compounds have long been known to provide anodic inhibition to steel in the presence of oxygen (by promoting the formation of a protective oxide layer), and have been shown to provide a degree of cathodic inhibition in the presence of divalent cations (Ca^2+^, Zn^2+^), due to the precipitation of insoluble phosphate complexes^4,7–9^. In the case of polyphosphate species, inhibition is comparatively enhanced, although anodic protection appears to occur via a comparable mechanism^10–12^. One explanation lies in improved cathodic inhibition, provided by the formation of a tightly bound protective film. Whilst the presence of such a film was first described by Uhlig in 1955^13^, and has been confirmed by numerous authors since^10,14,15^, its formation is not well understood, since polyphosphates are expected to revert to orthophosphates by hydrolysis under the alkaline conditions at cathodic sites. Nonetheless, it is widely accepted that film quality determines inhibitor efficiency at the cathode.

In this contribution we examine early cathodic film formation in detail using a commercially available strontium aluminium polyphosphate (SAPP) inhibitor. A split galvanic cell, modelled on the cut edge of galvanised steel is used throughout, allowing the cathodic inhibition mechanism to be studied independently of any anodic processes. The early stages of film deposition are monitored using EIS and *in-situ* AFM, and then analysed with EDX and the recently developed AFM-IR technique.

## Methods

### Sample Preparation

Model cut edge cells were prepared by affixing 1 cm^2^ pieces of carbon steel (SAE 1008/1010, 0.7 mm thickness) to zinc sheet (0.3 mm thickness, Goodfellow) using araldite resin (Araldite 3138 and Arudur 3140, Huntsman), thereby creating an insulating layer between the metals. Once cured, these were then either electrically connected by soldering on one side (for AFM experiments), or attached to wire (for electrochemical testing) and the whole ensemble was mounted at 90° and set in araldite resin. The metallic samples were then exposed at the non-connected side by sequential grinding using incremental grades of silicon carbide abrasive discs up to and including 1200 grade.

### Electrochemical Testing

Electrochemical tests were performed in saturated strontium aluminium polyphosphate (SAPP, Huebach) in electrolyte solution (3.5. % w/w NaCl). Typically for this class of inhibitor, SAPP was found to be sparingly soluble; measurements were obtained from saturated solutions containing < 50 ppm SAPP by weight when dissolved into 3.5 wt % NaCl electrolyte. A four-electrode cell was used and attached to an ACM Gill AC Weld Tester instrument, utilising the steel and zinc as two working electrodes, a saturated calomel reference electrode and a platinum counter electrode, as described in detail previously^3^. Impedance measurements were taken at the measured system coupled potential in the frequency range of 10,000 to 0.01 Hz with a signal amplitude of 10 mV.

### *In situ* AFM imaging

Cathodic film formation was examined in saturated (<50 ppm) SAPP in electrolyte solution (3.5. % w/w NaCl) using an atomic force microscope (Multimode 8, Bruker, Santa Barbara) equipped with a fluid cell at ambient temperature (26 °C). Images were collected in peakforce tapping mode using a Pt-Ir coated probe (nominal spring constant 2 N/m, nominal resonant frequency of 80 kHz, Bruker).

### Electron Microscopy

Scanning electron microscopy images were obtained using a Zeiss Ultra 55 field emission gun scanning electron microscope equipped with an Oxford Instruments EDX system. Energy Dispersive X-ray Spectroscopy (EDX) was performed using an accelerating voltage of 10 kV. Reported spectra are the average of 10 individual measurements obtained from an array on central regions of the steel cathode after 1 hour, 3 hours, 6 hours and 24 hours exposure of model cut edge cells to saturated SAPP in 3.5% w/w NaCl electrolyte.

### AFM-IR

*Ex-situ* nanoscale infrared analysis (AFM-IR) was performed on a NanoIR2 system (Anasys Instruments, Santa Barbara) operating with top-down illumination. During AFM-IR analysis, specimens were illuminated by a pulsed, tunable infrared source (optical parametric oscillator, 10 ns pulses at a repetition rate of 1 KHz, approximate beam spot size 30 µm). Sub-diffraction limit resolution was achieved by monitoring the deflection of an AFM probe in contact with the surface. This results from rapid transient thermal expansion of the material in contact with the probe tip in response to infrared absorbance^16^. The recorded AFM-IR signal is the amplitude of induced AFM probe oscillation, obtained after fast Fourier transform. This has previously been shown to correlate to infrared absorbance measured using conventional macroscopic FTIR^17^.

### FTIR

Bulk infrared spectra were obtained from 64 co-averages collected in ATR mode (single bounce diamond IRE) using a Fourier transform infrared (FTIR) spectrometer (Nicolet 5700 spectrometer, Thermo Electron Corp.) operating at 4 cm^−1^ resolution across the 500–4000 cm^−1^ range.

### X-Ray Diffraction

X-ray diffraction (XRD) was employed to identify the crystalline phases of the strontium aluminium polyphosphate powder pigments (SAPP). A X-ray diffractometer (Bruker D8 Advance) was used to obtain the diffraction pattern of the powder SAPP pigment. The diffractometer was fitted with a copper anode (Cu K_α_ X-ray, 1.54 Å) while the excitation conditions were 40 keV and 40 mA. The diffraction pattern was collected in the range of 2θ Bragg angles from 10° to 120° while the step size was 0.02^o^ per 4.5021 seconds. The diffraction pattern was analysed with the X’Pert HighScore Plus Software (Malvern Panalytical) and the crystalline phases of the SAPP pigment were identified with reference to the International Centre for Diffraction Data (ICDD) database.

## Results and Discussion

**Figure 1:**
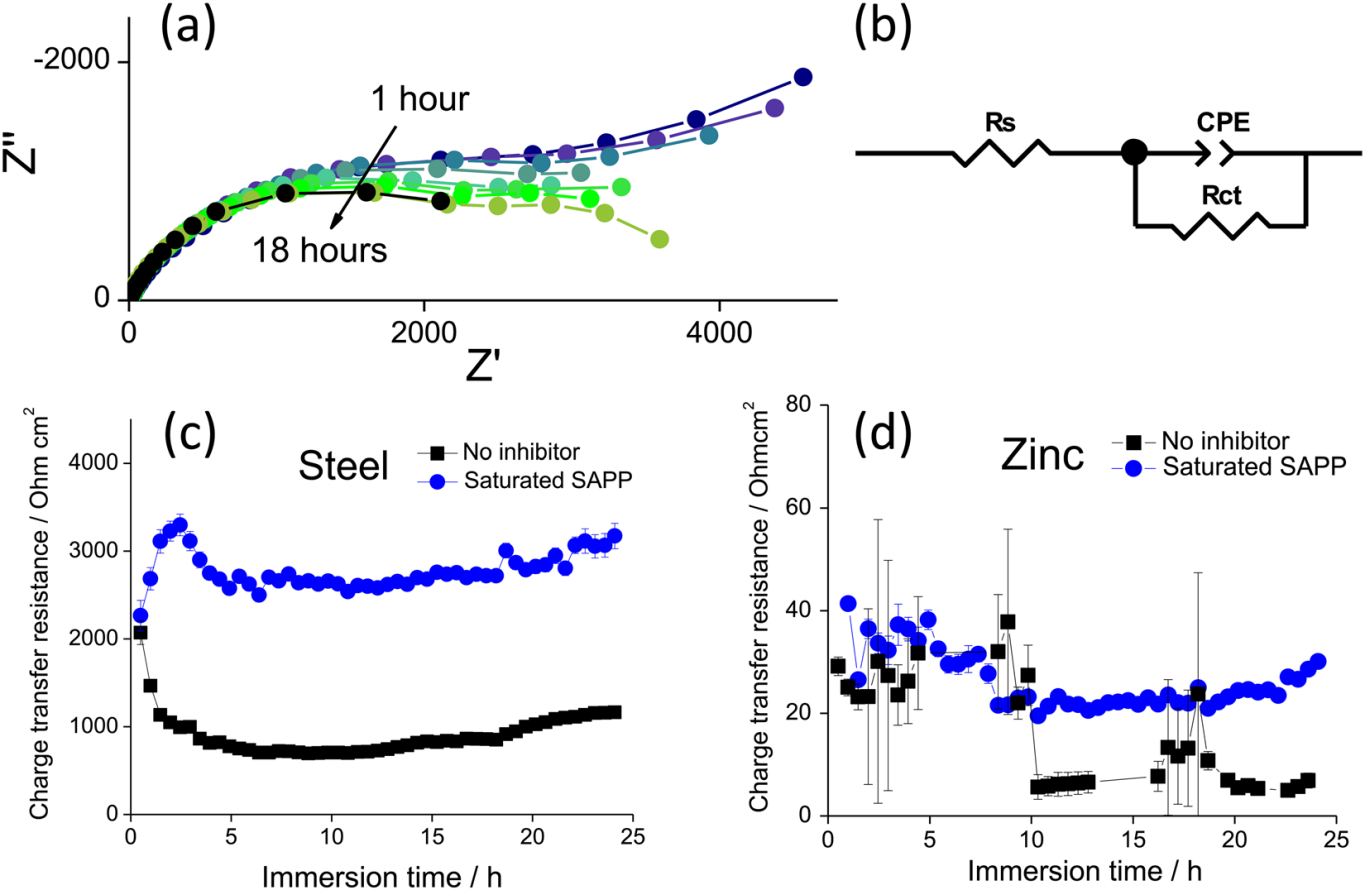
(**a**) Nyquist plots acquired for the steel part of a model cut edge cell after 1 hour, 1.5 hours, 2 hours, 2.5 hours, 3 hours, 3.5 hours, 4 hours, 4.5 hours and 18 hours immersion in saturated SAPP inhibitor in 3.5 wt % NaCl electrolyte (**b**) and the equivalent circuit used to fit data and calculate charge transfer resistance values for the (**c**) steel and (**d**) zinc electrodes as a function of time during 24 hours immersion.

In order to establish the individual contributions of anodic and cathodic inhibition by SAPP, a model split cell system comprised of carbon steel and zinc foil was employed for electrochemical impedance spectroscopy in the presence of a saturated SAPP solution, and compared to results obtained in the absence of any inhibitor. Spectra were monitored for the first 24 hours of immersion, and displayed single time constant behaviour that could be modelled using a simple equivalent circuit, Fig. 1.

Anodic and cathodic inhibition was evaluated by comparison of the charge transfer resistance values obtained through fitting the equivalent circuit to the impedance plots, Fig. 1^3^. It can be seen that inhibition occurs immediately (within 1 hour) at the steel cathode, whereas differences between the charge transfer resistance at the uninhibited and inhibited zinc anode were minor and relatively delayed.

Thus, these results confirm that cathodic film formation is the primary inhibition mechanism during the early stages of exposure to SAPP. This can be attributed to the clear films known to ultimately develop on cathodic regions in the presence of polyphosphate species^6,10,15^, however little is understood about the formation of such cathodic films. In the present work, fluctuations in charge transfer resistance values at the cathode are indicative of an evolving structure, where the formed film is broken down and replaced by an initially less resistive structure, which in turn grows increasingly protective.

**Figure 2:**
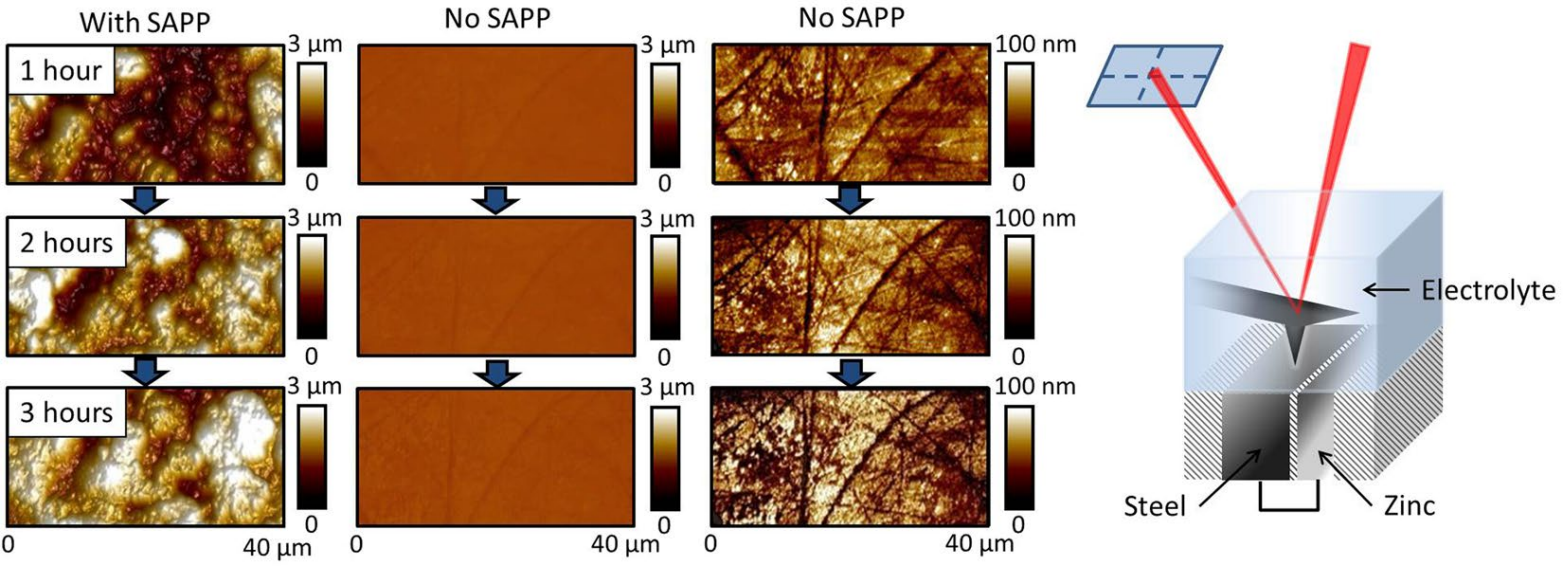
Peak force tapping mode AFM images of steel coupled to zinc during immersion in 3.5 wt % NaCl in the presence of a saturated solution (<50 ppm) SAPP inhibitor (left) and in the absence of added inhibitor, displayed using both a 3 µm and 100 nm z scale. The fluid cell experimental set up is also illustrated (right).

To confirm the presence of such a transient inhibiting film during very early exposure times, the cathodic steel was monitored *in-situ* using an AFM fluid cell, Fig. 2. In the presence of SAPP inhibitor, AFM surface profiles demonstrate that a thick gelatinous film indeed rapidly covers the steel electrode (within 1 hour), in keeping with the rapid inhibition evidenced by electrochemical tests. To confirm that this corresponds to an inhibitor film, an uninhibited control specimen was examined under identical conditions. In this case, only small crystalline deposits were observed (see images given at 100 nm z-scale, Fig. 2), corresponding to the zinc oxide and hydroxide species expected to form in the alkaline environment at the cathode^18^.

**Figure 3:**
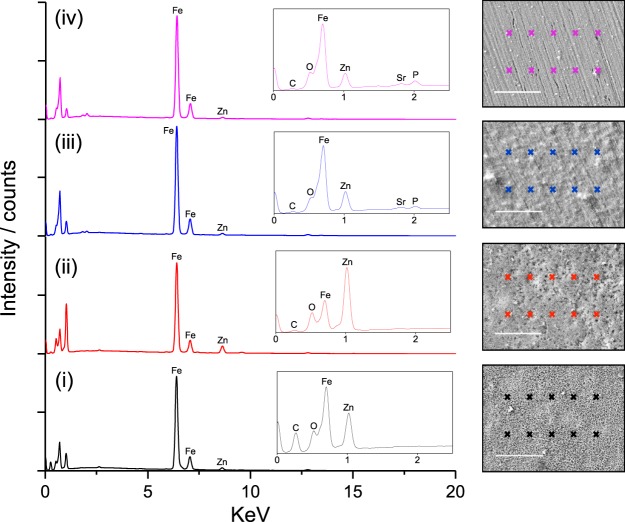
EDX spectra of the steel surface after (i) 1 hour (ii) 3 hours (iii) 6 hours and (iv) 24 hours immersion in a saturated solution (<50 ppm) SAPP inhibitor in 3.5 wt % NaCl. Insets show expanded 0–2.5 KeV regions. Spectra shown are the average of 10 individual measurements taken from regions of the specimens indicated by markers (right). Scale bar corresponds to 20 µm.

The evolving structure of cathodic films was further examined using *ex-situ* SEM and energy dispersive x-ray (EDX) analysis, Fig. 3. Note that in contrast to *in-situ* AFM analysis, the dehydrated films appear porous when examined under vacuum by conventional SEM. This disparity between *in-situ* and *ex-situ* morphology is important, since *ex-situ* SEM data has previously been used to assess inhibitor film structure^6,10,14^, and could lead to anomalous interpretations, e.g., that improving inhibition is a result of increasing surface coverage/decreasing porosity. EDX spectra were obtained from ten locations over the central region of the steel cathodes, and averaged for comparison, Fig. 3. This EDX elemental analysis demonstrated that the composition of inhibitor films evolved as a function of immersion time, in keeping with the electrochemical results.

Specifically, EDX analysis showed that all films contained zinc, but no strontium or aluminium was detected in the transient films formed up to 3 hours after immersion. This demonstrates that ion exchange with zinc released from the anode takes place before early deposition. More surprisingly however, phosphorous was also not incorporated into these early films, despite an increased local concentration of phosphorous frequently being cited as an explanation for these inhibitors superior performance in comparison to orthophosphate species^6,12,19^. After longer deposition times, some phosphorous is however detected.

**Figure 4:**
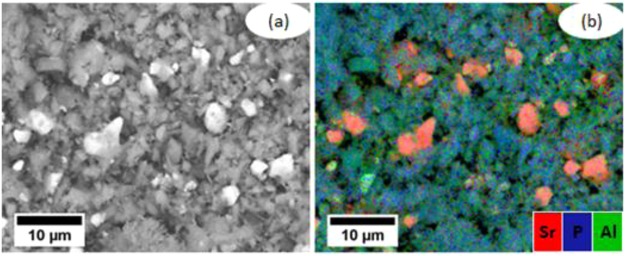
SEM micrograph and EDX maps obtained from the powder SAPP pigment: (**a**) the backscattered electron image of the powder SAPP pigment, and (**b**) the overlaid EDX elemental map of Sr, P and Al.

**Figure 5:**
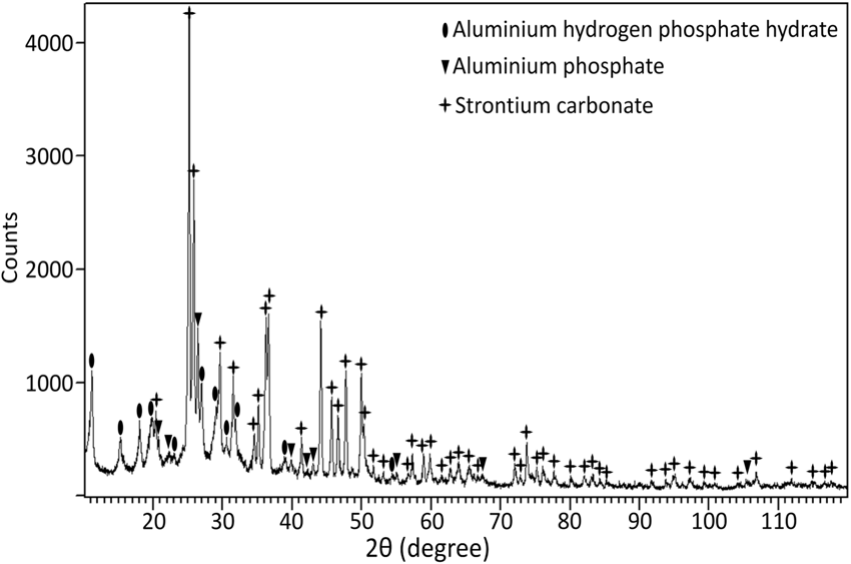
The X-ray diffraction pattern of the powder SAPP pigment.

These elemental compositions may partly be explained by the relative solubility of individual SAPP particles, since X-ray diffraction (XRD) and EDX measurements have shown that commercial SAPP is actually a heterogeneous mixture of strontium enriched SAPP particles, aluminium enriched SAPP particles, and impurities such as strontium carbonate and aluminium polyphosphate/hydrogen phosphate hydrate, Figs 4 and 5. Indeed, the EDX spectra of inhibitor films formed on steel after one and three hours of exposure to saturated SAPP solution indicate that the most soluble anion available, carbonate, is initially incorporated into the films.

**Figure 6:**
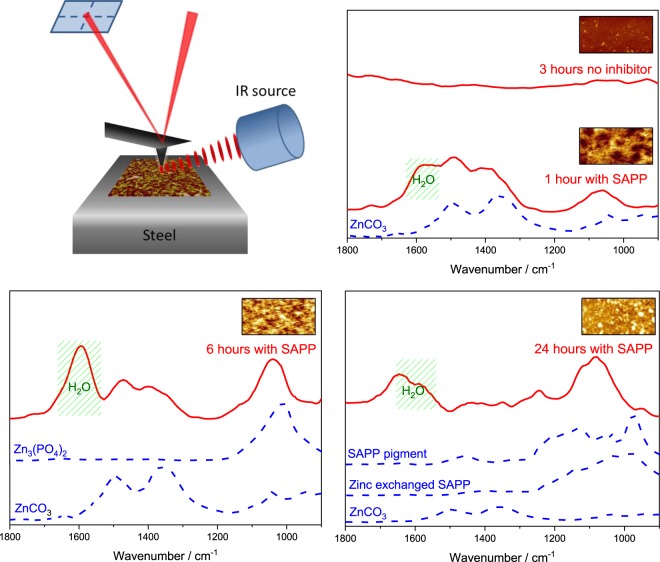
The AFM-IR experiment (top left); alongside AFM-IR spectra gathered from the steel cathode after immersion in 3.5 wt % NaCl with or without the addition of saturated concentrations of SAPP inhibitor (red solid lines). AFM-IR spectra correspond to the average of 200 individual measurements taken across the 40 µm x 20 µm regions shown in insets (z-scale is 2 µm).; Bulk ATR-FTIR spectra (blue dashed lines) are shown for comparison. For ATR-FTIR, ZnCO_3_ was precipitated from solutions of Na_2_CO_3_ and ZnCl_2_, Zn_3_(PO_4_)_2_ was precipitated from solutions of ZnCl_2_ and Na_3_PO_4_ at pH 11,and SAPP inhibitor was analysed both as received, and after ion exchange with ZnCl_2_.

To confirm this, further insight into the chemical structure of the cathodic films was achieved using the recently developed AFM-IR technique. In order to generate local infrared spectra from the thin films, transient expansion of the specimen was induced by exposure to 10 ns pulses of infrared radiation at wavenumbers corresponding to the vibrational excitation of polar bonds. This response is recorded by means of an AFM probe in contact with the surface, which is ‘kicked’ to vibrate by the specimen during photothermal expansion. To a first approximation, the amplitude of induced cantilever vibration is proportional to infrared absorbance at a given wavelength. Application of this technique, with spectral averaging, selectively yielded surface sensitive spectra from the thin films covering the steel, Fig. 6. In accordance with electrochemical and EDX results, the spectral signature of the SAPP-derived inhibitor films evolved as a function of exposure time. After 1 hour, spectra are dominated by the OH bend around 1600 cm^−1^ (due to residual water) and carbonate peaks (asymmetric v_3_ (CO_3_)^2−^ stretching bands at 1400 cm^−1^ and 1488 cm^−1^), indicating that the film is primarily composed of zinc carbonate species. Thus, it can be said that the initial stage of film formation involves the combination of dissolved strontium carbonate particles, previously thought to have no role in inhibition, and zinc ions diffusing from the anode. This is also expected to be the case for cathodic corrosion protection initially offered by the leachate of embedded SAPP pigment particles, since the strontium carbonate is more soluble than the other identified components of the inhibitor (whereas zinc carbonate is sparingly soluble)^20^.

After longer exposure times (>6 h), carbonate bands were still evident in spectra, but the OH bend (due to residual water) and a broad peak centred at 1060 cm^−1^ become relatively more intense. This is coincident with the detection of phosphorous by EDX, and corresponds to the position of the single infrared band measured for zinc orthophosphate when precipitated from alkaline solutions (V_3_ (PO_4_)^3−^, bending mode of fully deprotonated orthophosphate), Fig. 6. The presence of orthophosphates can be ascribed to the hydrolysis of P-O-P bonds in polyphosphates, which is known to occur under alkaline conditions^21^. This reversion to orthophosphate species at the cathode has been proposed^11^, but not confirmed, before, since local pH values are known to exceed 10 when corrosion at the cut edge is uninhibited^22,23^. Previously however, films attributed to polyphosphate species (morphologically distinct from the precipitates formed by orthophosphate inhibitors) have been detected in *ex-situ* tests after lengthy exposure times^15,19,24^. In accordance with those studies, polyphosphate absorbance peaks at 1244 cm^−1^ and 1352 cm^−1^ (corresponding to symmetric and asymmetric PO_2_ vibrations respectively) were in fact detected after 24 hours exposure^25^.

Taken in conjunction with the EDX data, it can be surmised that as the cathodic reaction becomes progressively inhibited, the local pH is lowered sufficiently to allow the precipitation of insoluble zinc and strontium polyphosphates. This means that precipitation of polyphosphate species can only occur once inhibition is active and the local pH is lowered. Active inhibition will, however, also coincide with a reduced rate of zinc ion release from the anode. The early formation of a thick zinc carbonate layer may therefore provide a secondary source of zinc via its slow dissolution, facilitating the deposition of more hydrolytically stable zinc (poly)phosphate species (note that whilst both zinc carbonate and zinc phosphates are considered sparingly soluble in aqueous media, the solubility product constants have been calculated to be 1.5 × 10^−10^ and 7.8 × 10^−37^ for ZnCO_3_ and Zn_3_(PO_4_)_2_ respectively, and zinc polyphosphates are expected to be even less soluble^26^). Note that, aside from the *in-situ* AFM analysis, all the experiments described here were performed in duplicate. Cathodic protection, accompanied by an increased phosphorus:carbon elemental ratio over the cathode, and the emergence of P-O-P infrared bands were all evident in repeats, alongside the presence of carbonate absorbance bands in early AFM-IR spectra.

To summarise, cathodic inhibition is shown to dominate at the galvanised steel cut edge in the presence of dissolved strontium aluminium polyphosphate (SAPP) inhibitor pigments. This is because a thick film rapidly precipitates onto cathodic regions (coverage is indicated after only one hour exposure using *in-situ* AFM measurements). EDX elemental analysis and the AFM-IR technique showed that the rapid formation of a cathodic film is in fact due to the presence of highly soluble strontium carbonate impurities in the commercially available pigment, which leads to the capture of zinc ions in the form of a zinc carbonate layer. Surprisingly, the initial coverage by zinc carbonate itself provides effective corrosion protection during early exposure times, before becoming an anchor for the eventual deposition of a dense, insoluble polyphosphate film. It is envisioned that this newly discovered synergistic effect could be used to improve formulation of these environmentally benign corrosion inhibitors.
